# Management of malignancy-associated bowel obstruction by cervical esophagostomy and total parenteral nutrition, case series of 2 patients

**DOI:** 10.1016/j.ijscr.2018.11.016

**Published:** 2018-11-16

**Authors:** Paul H. Sugarbaker, Puja G. Khaitan, Chukwuemeka Ihemelandu

**Affiliations:** a Center for Gastrointestinal Malignancies, MedStar Washington Hospital Center, 106 Irving St., NW, Suite 3900, Washington, DC 20010, USA; b Department of Surgery, MedStar Washington Hospital Center, Washington, DC, USA

**Keywords:** Intestinal obstruction, Cytoreductive surgery, Malignant ascites, Peritoneal metastases, Appendiceal cancer, Colorectal cancer, Case series

## Abstract

**Background:**

Patients with gastrointestinal or gynecologic malignancy may show disease progression within the abdomen and pelvis causing bowel obstruction in the absence of clinical evidence of disease outside of the peritoneal space. Bowel obstruction eventually results and has a profound effect on quality of life eventually leading to the demise of these patients.

**Methods:**

In order to improve the quality of life and combat the requirement for a nasogastric tube, a combination of total parenteral nutrition (TPN) to allow adequate caloric intake plus cervical esophagostomy to drain intestinal secretions was utilized as a palliative treatment long-term for patients with malignancy-associated bowel obstruction.

**Results:**

In 2 patients with appendiceal malignancy no cancer spread outside of the peritoneal space occurred. Unfortunately, total obstruction of the bowel caused unremitting nausea, vomiting, and pain accompanied by an inability to take adequate nutrition by mouth. In these 2 patients TPN long-term was initiated and a cervical esophagostomy tube placed percutaneously. Relief of malnutrition and the symptoms of nausea and vomiting were well treated using this combined approach. Pain management continued as an ongoing problem.

**Conclusions:**

Long-term management of malignancy-associated bowel obstruction is possible with improvement in quality of life using a combination of TPN and a cervical esophagostomy tube. In these two cases the cervical esophagostomy tube was placed with limited adverse events, and adequate drainage of intestinal secretions long-term.

## Highlights

• Progression of peritoneal metastases to cause bowel obstruction may occur in the absence of systemic cancer spread.
• Bowel obstruction has a profound effect on the quality of life of these patients prior to their demise.
• Drainage of gastrointestinal secretions has been shown to add palliative benefit to the management of these patients.
• Total parenteral nutrition can assist in improving the quality of life of these patients.
• In selected patients the optimal method for gastrointestinal decompression is a cervical esophagostomy tube.

## 1 Introduction

A large proportion of patients with gastrointestinal and gynecologic malignancy progress with the preponderance of their recurrent disease confined to the abdominal and pelvic space. Consequently, malignancy-associated bowel obstruction causing intolerable symptoms occurs prior to distant metastases. These patients fight to maintain oral nutrition at great expense to their quality of life in that repeated episodes of abdominal pain, nausea, and vomiting are brought about by their efforts to maintain oral intake. Currently, total parenteral nutrition is often used in the short-term to maintain patients with gastrointestinal dysfunction until oral nutrition can be reestablished by a reoperative procedure. Eventually, with few exceptions, patients with malignancy-associated bowel obstruction die as a result of starvation, intraabdominal abscess formation, or fistulization. The terminal events for these patients are invariably difficult and associated with extreme hardship for both the patient and their family [1,2]. Total parenteral nutrition (TPN) can be made available to these patients. However, in order for parenteral feeding to be well tolerated, gastrointestinal secretions need to be managed efficiently and safely. Very often a percutaneous gastrostomy tube can be placed in these patients with good long-term results [3]. Unfortunately, the extent of cancer within the abdomen, ascites, or prior surgery may cause difficult or impossible placement of a gastrostomy tube. In this group of patients, if prolonged TPN is contemplated, nasogastric suctioning is required to decompress the gastrointestinal tract. A long-term tube through the nose and into the stomach is not compatible with a tolerable quality of life.

In this report we collect our recent experience in 2 patients with a cervical esophagostomy tube for long-term gastrointestinal decompression as a result of malignancy-related bowel obstruction. Successful short-term management of these patients by TPN and a cervical esophagostomy tube can improve the quality of life of these patients. This case report is registered as a case series on the www.researchregistry.com website with UIN 4393.

## 2 Methods

The disease process causing gastrointestinal obstruction including clinical management, radiology, abdominal surgery, and pathology were reviewed. The rationale for a palliative intervention was established. The surgical procedure to provide intestinal decompression and the use of intravenous nutrition were described. Data on these patients was prospectively recorded at an academic institution. All information was from a single institution and the cases were consecutive. This research work has been reported in line with the PROCESS criteria [4].

## 3 Patient presentations

### 3.1 Patient 1

A 60 year old emergency medicine physician had surgery for appendicitis 06/23/2008. The laparoscopic appendectomy revealed an adenocarcinoma. On 07/23/2008, he was taken back to the operating room for a right colon resection. There was no residual tumor within the specimen and 26 mesenteric lymph nodes were free of cancer. In February of 2015, he had a bloody bowel movement. On 03/05/2015, a colonoscopy showed adenocarcinoma in and around the right colectomy site. CT showed mucinous adenocarcinoma infiltrating the omentum, mesentery of the right colon and rectosigmoid colon with a small amount of disease covering the peritoneal surface beneath the right hemidiaphragm.

**Fig. 1:**
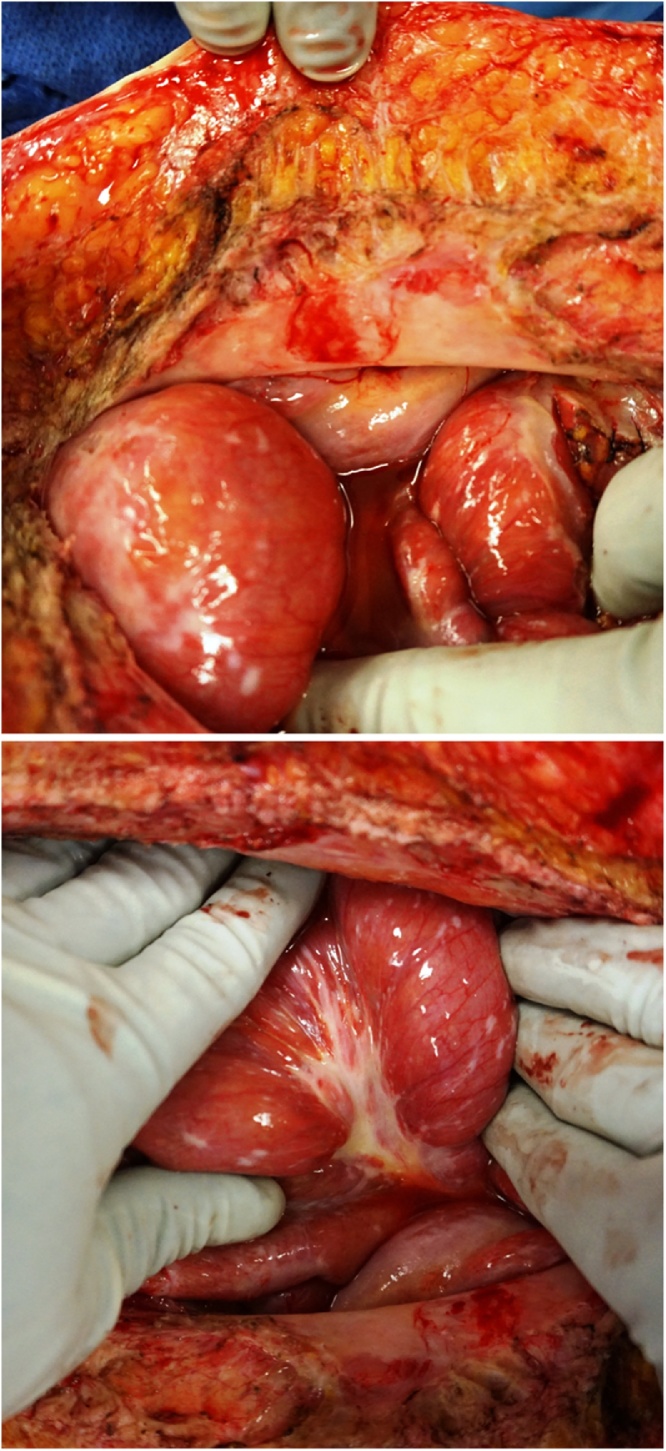
Top. At the lower aspect of the abdominal incision the parietal peritoneum is opaque indicating an infiltration by cancer. The loop of small bowel at the lower aspect of the abdominal incision shows a seromuscular layer infiltrated by the mucinous adenocarcinoma. Bottom. This loop of bowel shows numerous infiltrative cancer implants within the seromuscular layer. A dense infiltration of the cancerous process is layered out on the mesentery of this loop of small bowel.

Chemotherapy with FOLFIRI and Erbitux was used for 12 cycles with a marked decrease in tumor volume. He was taken to the operating room for cytoreductive surgery on 10/01/2015 where he had a 9-h surgical procedure which required peritonectomy of the right upper quadrant, greater omentectomy, pelvic peritonectomy and rectosigmoid colon resection with anastomosis [5]. He received hyperthermic intraperitoneal chemotherapy (HIPEC) with doxorubicin, mitomycin C, intravenous 5-fluorouracil, and leucovorin [6]. In June of 2016, he began with intermittent episodes of intestinal obstruction treated by nasogastric suctioning and TPN. His CEA tumor marker had increased to 30 ng/ml. CT scans were interpreted as normal. He was taken back to the operating room for exploratory laparotomy on 03/01/2018. Surgery revealed a contiguous infiltration of both parietal and visceral peritoneum by mucinous adenocarcinoma with no obvious sites of intestinal obstruction (Fig. 1). The encasement of the large and small bowel by cancer had resulted in loss of gastrointestinal peristalsis. Gastrostomy tube placement was not possible because of fixation of the stomach by cancer high in the left upper quadrant.

Postoperatively, the patient was maintained on TPN and nasogastric suctioning. The nasogastric tube could not be removed because of a high volume of enteric contents suctioned on a daily basis.

On 03/07/2018, a silastic 18 French tube was placed percutaneously as a cervical esophagostomy by an experienced thoracic surgeon (PGK) without incident (Salem silicone dual lumen stomach tube, Covidien, Mansfield, MA) [7]. Initially, a kinking of the distal portion of the tube occurred which interfered with gastric drainage. The esophagostomy tube was repositioned and functioned well. The patient has now been maintained on parenteral feeding and esophagostomy tube suctioning for 4 months with good results. Pain management from abdominal distention remains a problem. Follow-up by phone and review of radiology reports has continued until 07/16/2018.

### 3.2 Patient 2

**Fig. 2:**
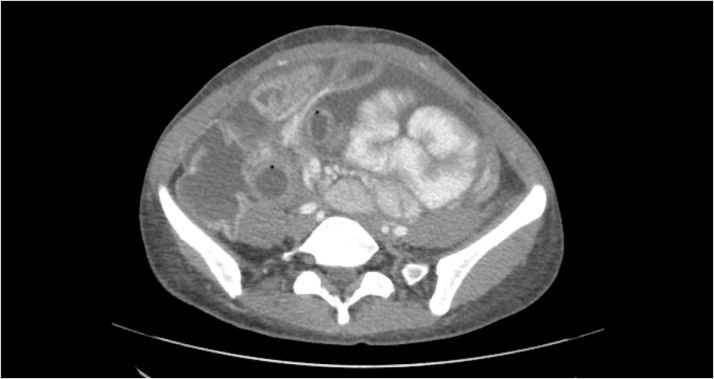
CT from March 1, 2018 shows extensive ascites with small bowel clumped from mesenteric retraction by cancer.

This 39 year old woman presented in 2015 with abdominal pain and distention. By CT and biopsy, a diagnosis of signet ring adenocarcinoma of the appendix was made. She was taken for cytoreductive surgery plus HIPEC with mitomycin C on 10/23/2015. Following this she was treated with 12 cycles of FOLFOX chemotherapy. In the fall of 2017, she began noting abdominal tightness. In March 1, 2018, CT scans showed extensive ascites with tethering of the small bowel within the pelvis (Fig. 2). She experienced a 20 lbs weight loss. It was thought that her partial intestinal obstruction was due, at least in part, to the tense ascites. In March of 2018, she underwent multiple paracenteses followed by a single cycle of intraperitoneal chemotherapy with 5-fluorouracil. There was transient relief of symptoms but abdominal pain and vomiting recurred which led to an exploratory surgery on 03/14/2018. The abdomen was found to be frozen and gastrostomy placement was impossible because the stomach was fixed within the left upper quadrant as a result of her prior extensive cytoreductive surgery. Postoperatively, she required continuous nasogastric suctioning. On 03/26/2018, she underwent percutaneous placement of an esophagostomy tube by an experienced thoracic surgeon (PGK) [7]. A left-sided pneumothorax that occurred with the tube placement was treated by a chest tube. Throughout her hospitalization and on hospital discharge she was maintained on TPN. Intermittent pain was controlled with a Fentanyl transdermal patch (Janssen Pharmaceuticals, Inc., Titusville, NJ). This symptom management and nutritional support has continued for 4 months without incident while the patient searched for further treatment options. The patient died as a result of progressive disease on 05/02/2018.

## 4 Discussion

This combined use of TPN for nutrition and gastrostomy or esophagostomy tube for control of gastrointestinal secretions is a departure from a traditional management of patients with malignancy-associated gastrointestinal cancer. Although placement of the esophagostomy tube is inexpensive and associated with a low incidence of complications the parenteral feeding long-term is expensive. However, a reasonable comparison is the expense in these patients to that required for ventricular-assist devices placed long-term for heart failure. Also compare the cost of long-term TPN to that required for long-term dialysis for chronic renal failure. An estimate of the cost of a year of parenteral feeding in the Washington, DC area is $100,850.

Previous investigators have suggested that prolonged intestinal feeding or decompression through an esophagostomy tube is possible. Yoon and colleagues studied 15 patients who underwent this procedure over a four year time period [8]. Their most common adverse event was accidental tube dislodgement. A single patient developed a tracheal esophageal fistula. No procedure-related mortality was seen and a majority of their patients were able to be discharged from the hospital. Rotter and Stuck reported the cervical esophagostomy of help in the management of 12 patients with severe dysphagia after radiation therapy for pharyngeal cancer [9]. No serious adverse events were reported. One of our 2 patients developed a pneumothorax with placement of the esophagostomy tube.

The veterinary literature contains numerous reports of esophagostomy feeding in dogs and cats. The procedure for tube placement is generally different with a tube placement from within the esophagus out through the neck skin [[10], [11], [12]].

The use of a cervical esophagostomy tube in patients with malignancy-associated bowel obstruction may be currently underutilized. Lilley and colleagues showed in a large number of patients with pancreatic or gynecologic malignancy that a gastrostomy tube is associated with improved patient management [3]. There are, however, many reasons why individual patients cannot have a percutaneously-placed gastrostomy. Cervical esophagostomy should be considered as an alternative to gastrostomy in this group of patients.

Currently, there are some deficiencies in the cervical esophagostomy management of malignancy-associated bowel obstruction. No tube has been specifically designed for cervical esophagostomy. The tube should be silastic [9,11]. The tube should not protrude extensively from the neck. The tube should not require sutures to the skin of the neck. It should be managed by a collar on the tube at the skin level that is secured with an umbilical tape around the neck. Routine management of the skin exit site of the tube needs to be performed in order to keep the skin exit site clean and free of irritation or granulation. The suction apparatus required for optimal maintenance has not yet been described.

The first exchange of the tube is important. It should probably be carried out under fluoroscopy and then the tube changed on a three monthly basis. This is to keep it from becoming coated internally by concretions and losing its efficiency as a suction drainage tube.

In some patients it has been observed that prolonged and adequate gastrointestinal decompression may facilitate some return of bowel function as the gastrointestinal tract is decompressed. Decompression may relieve acute angulation of bowel loops with partial return of bowel function. The possibility for limited oral intake that may be possible with patients with esophagostomy tube has not yet been clearly defined. Intermittent clamping with some nutrition within the stomach and upper small bowel may be possible.

The percutaneous cervical esophagostomy insertion using a Seldinger technique under endoscopic control can be performed with limited adverse events [7]. A larger series of patients is necessary before general comments regarding the safety of the procedure can be established. One of our two patients required a chest tube for pneumothorax that resulted from this procedure. Liang and colleagues described a procedure performed under general anesthesia. A 21 gauge introducer needle from a micropuncture introducer set (Cook Medical, Bloomington, IN) is inserted anterior to the sternocleidomastoid muscle. When the needle is seen by endoscopy to be within the esophagus, the guide wire from the micropuncture introducer set is threaded deep into the esophagus. The tract is then dilated using an introducer needle. Ultimately, the tract from skin to esophagus is dilated to the size of the desired tube. A peel away sheath (Kimberly Clark, Rosswell, GA) is used for placement of the silastic tube. The tube is guided into the stomach under direct endoscopic visualization. The tube is anchored at the neck. Alternatively, as described by Toh Yoon and Nishihara, the tube can be inserted under ultrasonographic guidance with the use of a rupture-free balloon [8].

In summary, an alternative method for gastrointestinal decompression in patients who are not candidates for gastrostomy was described. The cervical esophagostomy combined with total parenteral nutrition can assist in improving the quality of life of patients with malignancy-associated bowel obstruction in the absence of systemic cancer dissemination. The indications and results of cervical esophagostomy tube placement in two patients were presented.

## Conflicts of interest

The authors have no conflicts of interest to declare.

## Sources of funding

Secretarial support and data management support funded by Foundation for Applied Research in Gastrointestinal Oncology.

## Ethical approval

Local IRB-approval for this case report was not required:

MedStar Health Institutional Review Board has determined that a case report of less than three (3) patients does not meet the DHHS definition of research (45 CFR 46.102(d)(pre-2018)/45 CFR 46.102(l)(1/19/2017)) or the FDA definition of clinical investigation (21 CFR 46.102(c)) and therefore are not subject to IRB review requirements and do not require IRB approval.

This case report is only of 2 patients.

## Consent

Written and signed consents were obtained from the patients.

## Author contribution

Paul H. Sugarbaker, MD: study concept or design, data collection, data analysis or interpretation, writing the paper

Puja G. Khaitan, MD: study concept or design, data collection, data analysis or interpretation, writing the paper

Chukwuemeka Ihemelandu, MD: study concept or design, data collection, data analysis or interpretation, writing the paper

## Registration of research studies

This case report is registered as a case series on the www.researchregistry.com website with UIN 4393.

## Guarantor

Paul H. Sugarbaker, MD.

## Provenance and peer review

Not commissioned, externally peer reviewed.
